# Icariin and Its Metabolites as Potential Protective Phytochemicals Against Alzheimer’s Disease

**DOI:** 10.3389/fphar.2019.00271

**Published:** 2019-03-19

**Authors:** Cristina Angeloni, Maria Cristina Barbalace, Silvana Hrelia

**Affiliations:** ^1^School of Pharmacy, University of Camerino, Camerino, Italy; ^2^Department for Life Quality Studies, University of Bologna, Bologna, Italy

**Keywords:** icariin, icaritin, icariside, phytochemicals, Alzheimer’s disease, oxidative stress, inflammation

## Abstract

Alzheimer’s disease (AD) is a neurodegenerative disorder affecting more than 35 million people worldwide. As the prevalence of AD is dramatically rising, there is an earnest need for the identification of effective therapies. Available drug treatments only target the symptoms and do not halt the progression of this disorder; thus, the use of natural compounds has been proposed as an alternative intervention strategy. Icariin, a prenylated flavonoid, has several therapeutic effects, including osteoporosis prevention, sexual dysfunction amelioration, immune system modulation, and improvement of cardiovascular function. Substantial studies indicate that icariin may be beneficial to AD by reducing the production of extracellular amyloid plaques and intracellular neurofibrillary tangles and inhibiting phosphodiesterase-5 activity. Moreover, increasing evidence has indicated that icariin exerts a protective role in AD also by limiting inflammation, oxidative stress and reducing potential risk factors for AD such as atherosclerosis. This mini-review discusses the multiple potential mechanisms of action of icariin on the pathobiology of AD including explanation regarding its bioavailability, metabolism and pharmacokinetic.

## Introduction

Alzheimer’s disease (AD) is a progressive irreversible neurodegenerative disease that is becoming a population aging-related concern for public health systems all over the world due to its direct and indirect costs (Dos Santos et al., 2018). Clinically, AD is mainly characterized by cognitive and memory decline and accounts for up to 70% of all dementia cases in the elderly (Hebert et al., 2003) affecting more than 35 million people worldwide (Povova et al., 2012). AD possesses a multifactorial etiology that involves different pathophysiological processes like abnormal protein aggregation, neurons and synapses degeneration, neuroinflammation, mitochondrial damage, oxidative stress and excitotoxicity, which interfere with several neurotransmitters signaling pathways (Behl and Ziegler, 2017).

In particular, two major hallmarks characterized AD: extracellular accumulation of amyloid β peptide (Aβ) and intraneuronal aggregation of tau protein also known as neurofibrillary tangles (NFTs) (Calderon-Garcidueñas and Duyckaerts, 2017). Aβ is synthesized in the brain by the cleavage of the transmembrane amyloid precursor proteins (APP). Two secretases are responsible for Aβ production: β-secretase activity cleaving enzyme (BACE1) and the γ-secretase complex. BACE1 cleaves APP, producing an APP C-terminal fragment, which is subsequently cleaved within the transmembrane domain by γ-secretase at 40 or 42 residues, leading to the release of two different Aβ peptides Aβ_1-40_ or the most abundant Aβ_1-42_ (Thal et al., 2015) due to the variability in the C-terminus of Aβ (Tamagno et al., 2005). When APP is catabolized by other enzymatic activities (α- and Z-secretase complexes), Aβ is not produced.

In the normal brain, tau has 2 or 3 phosphate groups and binds to microtubules through electrostatic interaction (Jho et al., 2010). In AD, tau becomes hyperphosphorylated and the phosphorylation alters the net charge affecting the conformation of the microtubule binding region, thereby causing detachment of tau from microtubules that accumulates inside the neurons and aggregate to form NFTs (Trojanowski and Lee, 2002).

Beside Aβ plaques and NFTs, more than 50% of AD patients exhibit concurrent α-synuclein pathology (Twohig et al., 2018). α-synuclein is a 140 amino-acid protein abundantly expressed in neuronal presynaptic terminals. Different studies suggest that α-synuclein might be involved in the development of AD from the very early stages of Aβ pathology formation (Uéda et al., 1993; Vergallo et al., 2018).

Two other recognized pathological features of AD are neuroinflammation and oxidative stress (González-Reyes et al., 2017). In the normal brain, microglia does not produce proinflammatory molecules or reactive oxygen species (ROS), but in AD, Aβ induces the activation of astrocyte and microglia with a sustained release of proinflammatory molecules (Yucesoy et al., 2006). Elevated brain concentrations of inflammatory cytokines such as interleukin-1α (IL-1α), IL-β, IL-6, and tumor necrosis factor-α (TNF-α) have been associated with AD (Zilka et al., 2006). It has been shown that brain tissues in AD patients are exposed to oxidative stress (Gella and Durany, 2009), a condition characterized by an imbalance between ROS production and the endogenous antioxidative defense system.

Another very common feature of patients with AD is vascular dysfunction (Iadecola, 2005). It has been observed that a reduction in cerebral blood flow leads to a decline of Aβ clearance from the brain promoting neuronal degeneration and onset of AD (Zlokovic, 2011). On these bases, it is very important to improve endothelial function to prevent/counteract AD. Phosphodiesterase-5 inhibitors might interfere with the pathophysiological processes of AD such as neurovascular dysfunction (Sabayan et al., 2010). In particular, they can exert their positive effect on learning and memory by activating the NO/cGMP pathway (Puzzo et al., 2009) that produces a regulatory effect on endothelial function by relaxing blood vessels (Schulz et al., 2002). Moreover, cGMP could be used as a secondary messenger of the neurotransmitter acetylcholine (de Vente, 2004). Consequently, the inhibition of phosphodiesterase-5 is considered a novel approach to prevent/counteract AD.

Nowadays, several therapeutic strategies are used in clinical practice to counteract AD, however, all the drugs utilized are not able to alt or slow AD progression and possess many side effects (Mancuso et al., 2011). Therefore, there is a great interest in exploring new potential drug candidates for the treatment of AD. Antioxidant and anti-inflammatory activities of phytochemicals have been widely reported (Angeloni and Hrelia, 2012; Tarozzi et al., 2013; Angeloni et al., 2015, 2017). In this background, nutraceuticals are interesting therapeutic compounds to be explored as preventive and beneficial agents for AD. Icariin is a flavonoid present in Herba Epimedii, a traditional Chinese herbal medicine. Icariin has been shown to possess several biological activities. This mini-review focuses on the role of icariin and its metabolites in AD. Substantial studies indicate that icariin and its metabolites may be beneficial to AD by reducing the production of extracellular amyloid plaques and intracellular NFTs and inhibiting phosphodiesterase-5 activity. Moreover, increasing evidence has indicated that icariin exerts a protective role in AD also by limiting inflammation, oxidative stress and reducing potential risk factors for AD such as atherosclerosis.

## Icariin

Icariin (molecular formula: C_33_H_40_O_15_, molecular weight: 676.67 g/mol) is a prenylated flavonoid considered as the main bioactive of Herba Epimedii, a traditional Chinese herbal medicine used since thousands of years. Giving its therapeutic effects such as osteoporosis prevention, ameliorating sexual dysfunction, modulation of immune system, and improvement of cardiovascular function, Herba Epimedii has been included into Chinese pharmacopeia, indicating icariin as quality marker (Committee, 2010; Tang and Eisenbrand, 2011).

In tradition, icariin has been evidenced to possess anti-inflammatory, antioxidant, antidepressant and aphrodisiac effects (Liu et al., 2004; Tang and Eisenbrand, 2011; Liu et al., 2015). In addition, several *in vitro* and *in vivo* reports show many pharmacological activities elicited by icariin.

In different animal models of osteoporosis icariin demonstrated significant osteogenic effects mediated by Wnt/β-catenin and bone morphogenetic protein (BMP) signaling pathways (Wei et al., 2011; Li et al., 2013, 2014). Moreover, a clinical trial, conducted in postmenopausal women, showed a positive effect of icariin on bone mineral density (Zhang et al., 2007). Preliminary research suggests that icariin could be useful for treating erectile dysfunction as it was active on cavernous smooth muscle cells (Ning et al., 2006). The most promising effect of icariin at cardiovascular level is the promotion of stem cell differentiation into beating cardiomyocytes which suggests its likely application in cardiac cell therapy or tissue engineering (Jin et al., 2010; Zhou L. et al., 2013; Zhou et al., 2014). Moreover, icariin has also been evaluated for prevention and treatment of thrombosis in atherosclerosis as it reduces platelet adhesiveness and aggregation besides a decrease in serum cholesterol (Zhang et al., 2013). Multiple studies have indicated that icariin has been found to be beneficial to cancer (Zhang Y. et al., 2014), rheumatoid arthritis (Sun et al., 2013), immune system (Li et al., 2011), liver disease (Lee et al., 1995), diabetic nephropathy (Qi et al., 2011), sedative (Pan et al., 2005) and so on. Icariin has been found to possess multiple neuroprotective effects: it improves survival and function of neurons (Guo et al., 2010; Li F. et al., 2010) and triggers their self-renewal through neural stem cells (Huang et al., 2014).

## Pharmacokinetics of Icariin

**FIGURE 1:**
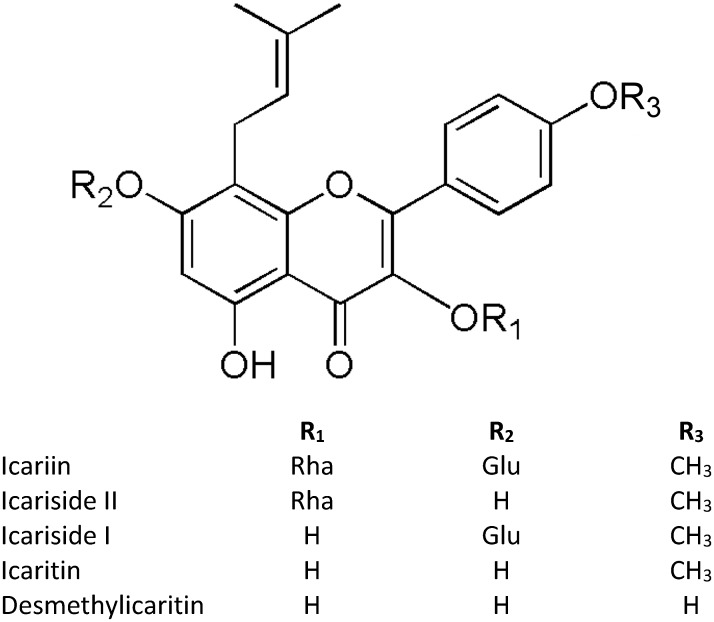
Structures of icariin and its metabolites. “Glu” refers to Glucose, “Rha” refers to Rhamnose.

Despite the numerous studies on icariin, the main challenge remains its very low oral bioavailability due to the physicochemical characteristics (Chen et al., 2008), and P-glycoprotein-mediated efflux in intestinal mucosa (Zhang Y. et al., 2012). Different studies indicated the importance of icariin hydrolysis by lactase phlorizin hydrolase in the small intestine and by microbiota β-glucosidase to release metabolites before its absorption (Zhao et al., 2010; Chen et al., 2011; Qian et al., 2012). In addition, icariin is a prenylated flavonoid and it has been reported that the prenyl-moiety decreases the bioavailability and plasma absorption of prenylated flavonoids (Chen et al., 2014). In this regard the presence of icariin in urine was less than 0.425% showing that probably the most of icariin is metabolized and excreted as metabolites (Figure 1) (Yu et al., 2016). Fortunately, the modern techniques offer a range of methods to overcome this issue. To increase icariin bioavailability, researchers have developed several drug delivery systems such as combining icariin with snailase (an exogenous hydrolase) to improve intestinal hydrolysis (Liu et al., 2017), encapsulating icariin into liposome (Yang et al., 2012), producing icariin/hydroxylpropyl-beta-cyclodextrin inclusion complex that enhances intestinal absorption probably through a solubilizing effect and/or the inhibition of P-glycoprotein (Zhang Y. et al., 2012).

Several methods have been used to investigate the pharmacokinetic characteristics of icariin and its metabolites like UFLC-TOF/MS (Qian et al., 2012), HPLC-MS/MS (Cheng et al., 2015; Sun et al., 2018), a liquid chromatographic method combined with electrospray ionization tandem mass spectrometry (Xu et al., 2017), and GC-MS (Shen et al., 2007).

After oral administration of *Epimedium* extract, the HPLC-MS/MS analysis of rat plasma revealed a rapid absorption and elimination of icariin, with a *t*_1/2_ ranged from 0.5 to 1 h; meanwhile the elimination of icariside II, which is chemically the monoglycoside form of icariin and *in vivo* predominant bioactive compound, from plasma takes a longer time from 3 to 18 h (Sun et al., 2018). Another study using a LC-MS method reported icariside II and icaritin (the aglycone form of icariin) as the major metabolites of icariin in rat feces after both oral and intramuscular administration (Xu et al., 2017). Interestingly, analyzing the data from various tissues (liver, heart, spleen, lung, kidney, brain, testicle, uterus and ovary) of male and female rats, the distribution of icariin differs in total tissue concentration (much higher in male rats than female rats) with the exception of genital organs (higher in females) (Xu et al., 2017). Interestingly, the pharmacokinetic profile of pure icariin depends on the route of administration. After oral administration, icariside II is the main form in rat plasma as 91.2% icariin is converted in it, but after intravenous injection only 0.4% of icariin is transformed in icariside II, demonstrating the role of intestinal microbiota in metabolizing icariin administrated *per o.s.* (Cheng et al., 2015). Indeed, it has been reported that icariin is metabolized to icaritin via icariside I and II by the rat intestinal microbiota (Zhou J. et al., 2013). Recently, a study on icariin metabolism by human microbiota evidenced a different pattern of metabolites depending on bacterial strains, and interestingly icariside I was not detected (Wu et al., 2016). In particular, the metabolites produced by human bacteria were icariside II, icaritin, and desmethylicaritin where the 4′-methyl of icaritin is removed. In human serum, the peak of icaritin was observed at 8 h after *Epimedium* decoction intake, suggesting that the conversion of icariin to icaritin is primarily at intestinal level, differently desmethylicaritin was not observed (Shen et al., 2007). Unfortunately, studies of icariin metabolism and distribution in humans are really few and should be improved to have a comprehensive view of the icariin pharmacokinetics properties. What emerged from these studies is that icariin is scarcely present in plasma because of its rapid elimination, and the tissue distribution of icariin in the brain is scarce, incoherently with the largely literature supporting neuroprotective effects (Xu et al., 2017; Zhang et al., 2017). Therefore, a possible explanation to this controversial issue could be that the observed biological effects are, in part, mediated by icariin metabolites. However, it is important to further improve the knowledge of the possible effects and mechanisms of icariin metabolites at cerebral level and against AD, and to develop and characterize novel delivery systems to increase the uptake and distribution of icariin in the brain.

## Icariin and Its Metabolites in Aβ Neurotoxicity

The effects of icariin in counteracting Aβ deposition and Aβ induced neurotoxicity have been largely investigated. The first study that reported an effect of icariin on Aβ was carried out in rats challenged with Aluminum (Luo et al., 2007). In particular, icariin (60 and 120 mg/kg) administrated by gavage for 3 months significantly attenuated Aβ_1-40_ production induced by Aluminum treatment. Besides, icariin counteracted learning and memory deficit, increased SOD activity and decreased MDA levels. These findings were further deepened by the same authors in a different AD model (Nie et al., 2010). In rats treated with Aβ_25-35_, icariin improved the learning and memory deficits by both a decreased production of insoluble fragments of Aβ due to the downregulation of β-secretase expression (BACE1) and to its antioxidatant activity. In the same experimental model, icariin nearly completely suppressed the abnormal inward calcium currents induced by Aβ_25-35_ in a dose dependent manner suggesting a potential neuroprotective effect of icariin on Aβ_25-35_-induced neurotoxicity via the balance of intracellular calcium homeostasis (Li L. et al., 2010). Urano and Tohda (2010) showed that icariin (50 μmol/kg) administrated for 8 days was effective in improving spatial memory impairment in 5×FAD rats, an AD model characterized by an elevated production of Aβ_1-42_ (Oakley et al., 2006). In a rodent APP/PS1 model of cerebral amyloidosis for AD a icariin (100 mg/kg by daily gavage) treatment for 10 days significantly attenuated Aβ deposition and restored impaired nesting behavior (Zhang Z. Y. et al., 2014). Zhang L. et al. (2014) demonstrated that icariin (100 μmol/kg for 6 months) counteracted Aβ burden and deposition in the hippocampus of APPV7171 transgenic mice by reducing the expression of both APP and BACE1. In the same experimental model, icariin (30, 60 mg/kg twice a day for 4 months) improved learning and memory of APP/PS1 mice in Y-maze tasks, reduced Aβ deposition, and down-regulated both APP and (phosphodiesterase-5) PDE5 (Zhang et al., 2010). Of note, the inhibition of PDE5 stimulated the NO/cGMP signaling pathway as evidenced by an increased expression of three nitric oxide synthase (NOS) isoforms, together with increased NO and cGMP levels in the hippocampus and cortex of mice. Similar results were obtained by Li F. et al. (2015) in Tg2576 mice treated with icariin (60 mg/kg) for 3 months. Icariin improved spatial working memory, reduced the levels of both Aβ_1-40_ and Aβ_1-42_, downregulated APP expression and enhanced neurogenesis. These aspects were further investigated using a triple-transgenic mouse model of Alzheimer’s disease (3× tg-AD) (Chen et al., 2016). An icariin treatment (65 mg/kg) for 6 months enhanced neuronal cell activity as identified by an increase of brain metabolite N-acetylaspartate and ATP production, preserved the expressions of mitochondrial key enzymes such as cytochrome c oxidase subunit 4 (COX IV) and pyruvate dehydrogenase E1 component subunit alpha (PDHE1α), and postsynaptic density protein 95 (PSD95), reduced Aβ plaque deposition in the cortex and hippocampus, and down-regulated BACE1 expression. Intragastric administration of icariin reversed the decreases in PSD95, brain derived neurotrophic factor (BDNF), pTrkB, pAkt, and pCREB expressions induced by Aβ_1-42_ injection in rats suggesting that icariin may improve synaptic plasticity through the BDNF/TrkB/Akt pathway (Sheng et al., 2017). The ability of icariin to increase pCREB was also observed in a senescence accelerated prom mouse model (SAMP8) characterized by early Aβ deposition (Zhang Z. et al., 2012). Moreover, icariin decreased the level of Aβ in rat hippocampus subjected to permanent occlusion of bilateral common carotid arteries (BCCAO) (Li W. X. et al., 2015), a model used to mimic cerebral hypoperfusion that occurs in vascular dementia and Alzheimer’s. This reduction of Aβ deposition was related to different effects such as the down-regulation of APP and BACE1, and an increased expression of insulin-degrading enzyme (IDE) and disintegrin and metalloproteinase domain 10 (ADAM10) in rat hippocampus. In an *in vitro* model, icariin (40–160 μg/mL) was able to dose-dependently protect cortical neurons against Aβ_1-40_ induced damage by enhancing the expression of CART and activating ERK signaling pathway (Sha et al., 2009). In addition, icariin 0.01 μM was able to counteract the axon and dendritic shortening induced by Aβ_1-42_ in rat cortical neurons (Urano and Tohda, 2010). In cultured rat PC12 cells icariin (20 μM) counteracted apoptosis induced by Aβ_25-35_ and this effect appeared to be mediated by the activation of the PI3K/Akt signaling pathway (Zhang et al., 2015). In agreement with these results, Zeng et al. (2010b) observed that icariin (5–20 μM) dose dependently reduced cell death and apoptosis in PC12 cells exposed to Aβ_25-35_. In addition, the authors demonstrated that this protection is partially due to activation of the PI3K/Akt signaling pathways that induces the inhibition of GSK-3β and, consequently, reduces tau protein hyperphosphorylation. Icaritin, another compound extracted from Epimedium, demonstrated to be neuroprotective against the toxicity induced by Aβ_25-35_ in primary rat cortical neurons (Wang et al., 2007). In particular, icaritin increased cell viability and reduced apoptosis by an estrogen receptor dependent mechanism and by activating ERK1/2 MAPK pathway.

## Icariin and Its Metabolites in Oxidative Stress

Oxidative stress plays a crucial role in the pathogenesis of many neurodegenerative diseases including AD. The antioxidant activity of icariin has been demonstrated in primary cortical neurons exposed to H_2_O_2_ (Zhang et al., 2010). In particular, icariin (1.2 μM) counteracted H_2_O_2_-induced neurotoxicity by reducing ROS production, increasing mRNA expression of the antioxidant enzymes catalase and peroxiredoxin 1 (PRX1) by a mechanism mediated by SIRT1 up-regulation. Icariin (5-50 μM) attenuates LPS-induced oxidative stress in primary microglial cells reducing ROS level in a dose dependent manner (Zeng et al., 2010a). In an *in vivo* study carried out in rats, icariin showed a protective effect against learning and memory deficit induced by aluminum by increasing SOD activity and decreasing malondialdehyde (MDA) levels (Luo et al., 2007). It has been shown that iron overload is involved in the progression of AD (El Tannir El Tayara et al., 2006). Excessive iron levels lead to increased oxidative stress through the Fenton reaction (Rolston et al., 2009). In order to counteract iron overload, APP/PSI mice were treated with icariin (120 mg/kg) for 3 months. Icariin reduced iron overload and protected mice against oxidative stress reducing lipid peroxidation and increasing the activity of the antioxidants enzymes SOD and glutathione peroxidase (Zhang et al., 2018).

Icariside also demonstrated to be effective in counteracting oxidative stress. Icariside II attenuated Aβ_25-35_-induced intracellular and mitochondrial ROS generation in PC12 cells (Liu et al., 2018).

## Icariin and Its Metabolites in Neuroinflammation

As previously underlined, AD is associated with neuroinflammation, which is triggered by microglia activation in the brain (Heneka et al., 2015). It is now widely accepted that these brain cells are likely to contribute to the mechanisms of neuronal damage and cognitive loss (Sarlus and Heneka, 2017). Icariin has been reported to have an anti-inflammatory effect on primary rat microglial cultures activated by LPS (Zeng et al., 2010a). In particular, icariin (5–50 μM) reduced the release of nitric oxide (NO), prostaglandin E (PGE)-2 in a dose dependent manner and down-regulated the expression of proinflammatory cytokines such as tumor necrosis factor (TNF)-α, interleukin (IL)-1β and IL-6. Icariin also inhibited the protein expression of inducible nitric oxide synthase (iNOS) and cyclooxygenase (COX)-2. The authors showed that the mechanisms behind this anti-inflammatory effect is the inhibition of the TAK1/IKK/NF-κB and JNK/p38 MAPK pathways. The ability of icariin (100 mg/kg for 10 days) to counteract microglia activation was also observed in the cortex and hippocampus of APP/PSI mice (Zhang Z. Y. et al., 2014) and these data were corroborated by a recent study of Zhang et al. (2018) that showed that icariin (120 mg/kg for 3 months) reduced neuroinflammation in the cerebral cortex of APP/PSI transgenic mice inhibiting the release of IL-6, IL-1β and TNF-α.

In an AD model obtained by ICV injection of STZ in rats, icariside II (10 mg/kg for 21 days) reduced the expression of TNF-α, IL-1β, COX-2, TGF-b1 by preventing the degradation of IkB-α and NFK-B p65 phosphorylation (Yin et al., 2016). These findings were confirmed by the results of Deng et al. (2017) showing that icariside II (20 mg/kg for 15 days) attenuated Aβ_25-35_-induced expression of TNF-α, IL-1β, COX-2, and iNOS in rat hippocampus.

## Conclusion

**FIGURE 2:**
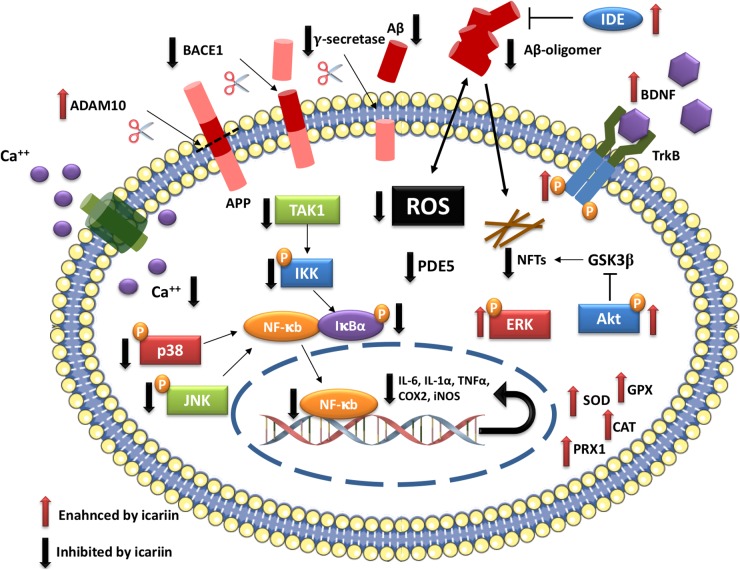
Icariin molecular targets in AD.

As reported, icariin, besides its use in complementary and alternative traditional Chinese medicine, is a very promising molecule to counteract many pathophysiological processes of AD, having an impact on Aβ production and removal pathways, on oxidative stress mediated effects and on neuroinflammatory cascade (Figure 2).

The practical possibilities of AD prevention and counteraction with this pleiotropic compound should be further investigated in clinical studies and represent a challenge for future researches.

## Author Contributions

CA conceived the idea. CA and MB prepared the manuscript. SH reviewed the drafts and provided important information for the completion of this manuscript.

## Conflict of Interest Statement

The authors declare that the research was conducted in the absence of any commercial or financial relationships that could be construed as a potential conflict of interest.
